# Circular DNA enrichment sequencing reveals the viral/satellites genetic diversity associated with the third epidemic of cotton leaf curl disease

**DOI:** 10.1093/biomethods/bpab005

**Published:** 2021-03-25

**Authors:** Nasim Ahmed, Imran Amin, Syed Shan-e-Ali Zaidi, Saleem Ur Rahman, Muhammad Farooq, Claude Maurice Fauquet, Shahid Mansoor

**Affiliations:** 1 Agricultural Biotechnology Division, National Institute for Biotechnology and Genetic Engineering (NIBGE), Constituent College, Pakistan Institute of Engineering and Applied Sciences (PIEAS), Faisalabad 38000, Pakistan; 2 Global Cassava Partnership for the 21st Century, CIAT, Apdo. Aereo 6713, Cali, Colombia

**Keywords:** cotton leaf curl disease, geminiviruses, begomoviruses, begomovirus epidemics, resistance breaking and mutations

## Abstract

Cotton leaf curl disease (CLCuD) is the most important limiting factor for cotton production in Pakistan. The CLCuD passed through two major epidemics in this region with distinct begomoviruses/satellites complexes. Since 2015 the disease has again started to appear in epidemic form, causing heavy losses to cotton crop, which we termed as the “third epidemic”. We applied CIDER-seq (Circular DNA Enrichment Sequencing), a recently developed sequencing method for PCR-free virus enrichment to produce a full length read of a single circular viral genome coupled with Sanger sequencing to explore the genetic diversity of the disease complex. We identified a highly recombinant strain of *Cotton leaf curl Multan virus* and a recently evolved strain of *Cotton leaf curl Multan betasatellite* that are dominant in all major cotton growing regions in the country. Moreover, we also identified multiple species of alphasatellites with one distinct species, *Mesta yellow vein mosaic alphasatellite* (MeYVMA) for the first time in cotton. Relative abundance of virus and associated satellites was also determined by real-time quantitative PCR. To the best of our knowledge, this is the first study that determined the CLCuD complex associated with its third epidemic.

## Introduction

Cotton (*Gossypium hirsutum*) is the most important fiber producing crop for many countries in the world. It not only provides fiber, but also plays a vital role in the oil and feed industries with its seed rich in oil and protein. China, India, USA, and Pakistan are the major cotton producing countries in the world [1]. In the Indian subcontinent, cotton is facing a real threat due to cotton leaf curl disease (CLCuD) [2]. The disease is caused by whitefly (*Bemisia tabaci*)-transmitted single-stranded (ss) DNA viruses of the genus *Begomovirus* that belong to the family *Geminiviridae* [3–5]. Begomoviruses are further divided into two groups; monopartite and bipartite based on their genomic components. The genomes of monopartite and DNA-A components of bipartite begomoviruses encode six proteins, two in the virion-sense orientation and four in the opposite orientation. In the virion-sense orientation, they encode the coat protein (CP) and V2/AV2 protein whereas in the dsDNA form they encode the replication-associated protein (Rep), the transcriptional activator protein (TrAP), the replication enhancer protein (REn), and the C4 protein on the complementary strand [6]. DNA-B components encode the two proteins, i.e. nuclear shuttle protein (NSP) and movement protein in the virion and complementary sense, respectively. The intergenic region (non-coding region) of begomovirus genomes/genomic components contains *cis*-acting regulatory elements for gene expression. The intergenic region also contains small repeated sequences, known as “iterons”, which are sequence-specific binding sites for replication associated protein (Rep) and a predicted hairpin structure containing the nonanucleotide sequence TAATATTAC which is conserved among most geminiviruses. The iterons and hairpin together form the origin of replication (ori) for viral DNA replication. Both components of bipartite begomoviruses share a sequence, known as the common region (CR) that acts to maintain the integrity of the split genome, ensuring that the DNA-A-encoded Rep can initiate replication of the virion strands for both components [7]. The monopartite begomoviruses are mostly associated with ssDNA molecules (∼half the size of begomovirus components) known as betasatellites and alphasatellites [8]. The betasatellites encode a single gene in the complementary sense that codes for an ∼118 amino acids protein known as βC1. Betasatellites enhance the disease symptoms and may increase the accumulation of their helper begomoviruses in some host plants [9, 10]. This is likely due to the fact that βC1 is a suppressor of RNA silencing [11, 12]. Alphasatellites (previously known as DNA-1; [13]) are capable of autonomous-replication in permissive host plants and therefore are not strict satellites. However, they need their helper begomoviruses for insect transmission between plants and movement within plants [14, 15]. Although widespread in the Old World (OW), alphasatellites have also been identified in the New World (NW) in association with bipartite begomoviruses, in the absence of betasatellites [16, 17].

Cotton leaf disease was first recognized in the 1960s in southern Asia and has gone through four distinct phases—pre-epidemic, epidemic, resistance breaking (second epidemic), and post resistance breaking. Although we can only speculate about the causative agent in the pre-epidemic phase, much is known about the causative agent in phases after it. Each of the three phases for which information is available was/is associated with distinct viruses but all characterized by the presence of a single betasatellite species—*Cotton leaf curl Multan betasatellite* (CLCuMuB) [2]. The epidemic phase was associated with the presence of multiple begomovirus species, the most common of which were *Cotton leaf curl Multan virus* (CLCuMuV) and *Cotton leaf curl Kokhran virus* (CLCuKoV) [3]. Toward the end of the epidemic phase CLCuD almost entirely disappeared from cotton as resistant cotton varieties, developed by conventional breeding, came into widespread cultivation by farmers [18]. The beginning of the resistance breaking phase (second epidemic) started in the early 2000s with the appearance of a recombinant strain of CLCuKoV and CLCuMuV, i.e. CLCuKoV-Burewala (CLCuKoV-Bu). The resistance breaking strain was also associated with a recombinant version of CLCuMuB. This resistance breaking strain had the truncated TrAP [19, 20]. The post-resistance breaking phase has witnessed a slow shift from CLCuKoV-Bu to a situation more akin to that of the epidemic phase, with at least some of the earlier virus species/strains reappearing in cotton [21]. Although other begomoviruses, and even a mastrevirus (leafhopper-transmitted geminivirus), were sporadically reported in cotton during all the phases for which information is available, the disease was always associated with CLCuMuB which is essentially required for symptoms development [22].

**Figure 1:**
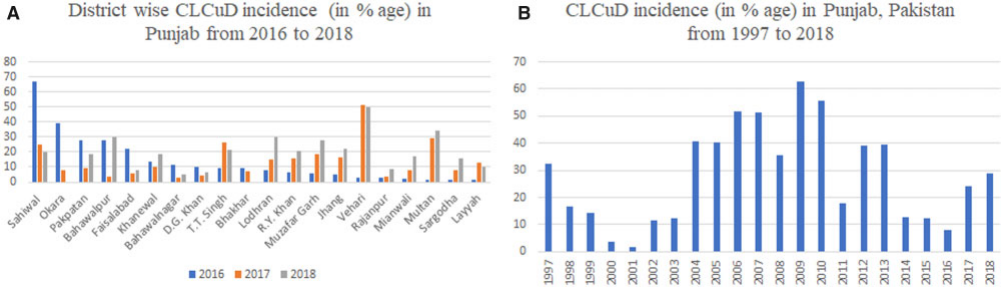
The incidence of CLCuD in Punjab province of Pakistan. (**A**) The district wise incidence of CLCuD in Punjab province of Pakistan during last 3 years (values are given in percentage). (**B**) The pattern of CLCuD in Punjab province from 1997 to 2018 (Data of CLCuD incidence were collected by “Agriculture extension department, Govt. of Punjab, Pakistan”).

**Figure 2:**
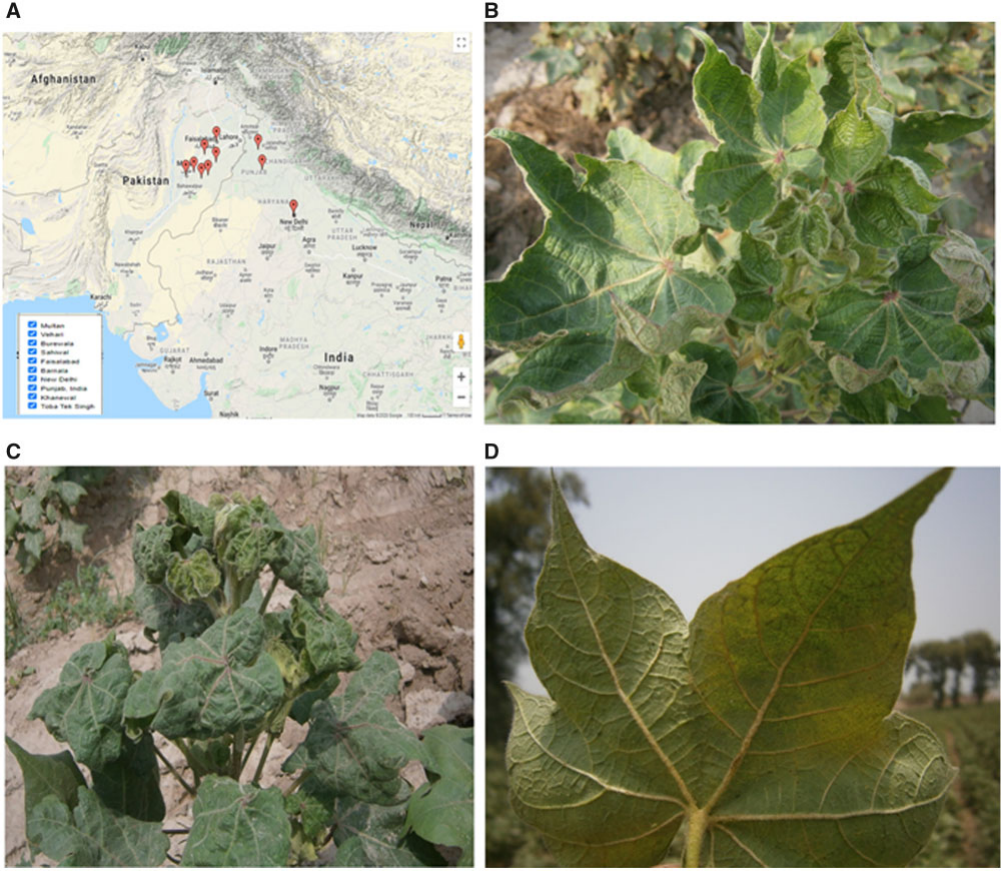
(**A**) Map showing the spread of this distinct strain of CLCuMuV in Indian sub-continent since 2015. Map was generated online on https://static.edit.g.imapbuilder.net/ by providing the names (listed on left bottom) of locations. (**B–D**) Typical symptoms of CLCuD observed in major cotton growing areas in current study; upward leaf curling (B), severe downward leaf curling and stunted plant growth (C), and vein thickening (D).

After 2014 there is a shift in the begomoviruses from CLCuKoV-Bu to the begomoviruses found in the first epidemic of CLCuD. In 2015, two independent studies carried out by Zubair *et al.* [21] and Datta *et al.* [23] in Pakistan and India, respectively, showed that they could not identify the CLCuKoV-Bu in infected cotton. Zubair *et al.* [21] found multiple begomoviruses which were associated with the first epidemic in 1990s whereas Datta *et al.* [23] exclusively found CLCuMuV in India from infected cotton in the fields. Their six isolates (three from each study) formed a novel clade in CLCuMuV. These two findings gave indications toward the evolution of the distinct strain of the CLCuMuV and its dominance in next epidemic in this region. A recent study [24] also predicted the third epidemic of CLCuD in this region based on the statistical data analysis and begomoviruses diversity in cotton in this region. They predicted the third epidemic in 2017–18 originated from Multan, Punjab, Pakistan. Their predictions of third epidemics actually occurred according to a predicted timeline. As field data of virus hotspots in the major cotton growing areas of Punjab, Pakistan were increasing in the year 2017–18 when compared with the last few years (Fig. 1). In this scenario, it was important to explore the genetic diversity of the CLCuD-associated begomoviruses (CABs) and satellites in this region. For this purpose, we designed a well-organized study to identify the begomovirus–satellite complex. In the July 2018, a survey of cotton fields was conducted in major cotton growing areas of Punjab, Pakistan. We visited more than 40 fields and observed the typical symptoms of CLCuD (Fig. 2) in majority of the fields and they were severely affected by CLCuD. We also observed the severe infestation of whitefly (*B. tabaci*) in all the fields. According to farmers, they sprayed the fields with insecticides many times but were unable to control whitefly and their fields were badly affected by CLCuD this year. We collected both symptomatic and non-symptomatic leaf samples from all CLCuD-affected fields. Using the standard protocols of our laboratory, the possible causative agents of the disease from infected leaf samples were amplified, cloned, and sequenced. Moreover, we applied a recently introduced new method; CIDER-seq (Circular DNA Enrichment Sequencing) [25] with some modifications to precisely determine the genetic diversity. This new technology overcomes the drawbacks/limitations of previously adopted sequencing methods and techniques. Previously used methods such as PCR with universal primers or restriction cloning and Sanger sequencing have the limitations of missing some molecules that may be associated with the disease. Secondly, next-generation sequencing (NGS) using the Illumina platform to sequence such molecules have the drawback of miss assembly of these begomoviruses (due to much recombination events among these viruses). So, the method used in the current has the advantage over previous methods by overcoming all these limitations. As Mehta *et al.* [25] applied this method for the detection of geminiviruses populations study in the transgenic plants but we applied in exploring the genetic diversity of complex natural begomoviruses/satellites in the field and developed the downstream analysis guidelines accordingly to make the overall adoptable method in other such complex diseases like chilli leaf curl disease, tomato leaf curl disease, etc. As due to mixed infections, begomoviruses got the opportunity to recombine with each other and result in the emergence of new strains or species. This is indicated by the largest number of species (>420) of begomoviruses so far reported.

In short applications of this method will be very helpful for precise identification of disease complex associated with other such diseases. Sequenced results showed the dominance of a distinct strain of CLCuMuV in association with CLCuMuB^Veh^ strain in major cotton growing areas of the Punjab. Moreover, we were able to find many species of alphasatellites. These results show the current status of begomoviruses–satellite complex associated with the third epidemic of CLCuD in this region.

Further, we explored the genetic variations in these new isolates of CLCuMuV-Raj strain to try and understand the actual changes evolved by this virus strain to become the dominant in new epidemic of CLCuD. In future, this information will be helpful in understanding the molecular mechanism of CLCuD by answering the question that why this CLCuMuV-Raj developed such specific mutations to overcome the resistance and became dominant over previously dominant strain CLCuKoV-Bu? In this scenario, it is important to understand the molecular players involved in the compatible interaction of virus and host to break this compatibility for durable and broad-spectrum resistant cultivars of cotton. Further, the importance of this newly developed method of sequencing the single molecule is discussed.

## Materials and methods

### Collection of the cotton samples infected with CLCuD

In July 2018, a survey of cotton fields was conducted in major cotton growing areas of Punjab, Pakistan. About 40–60% of most of the cotton fields were badly affected by CLCuD. Plants were showing the typical symptoms of CLCuD, i.e. upward and downward leaf curling, vein thickening, leaf enation, and stunted plant growth. We collected three symptomatic and two non-symptomatic leaf samples from each field.

### Total genomic DNA extraction and viral DNA enrichment

The total genomic DNA from both symptomatic and non-symptomatic leaf tissues was extracted using Cetyl trimethyl ammonium bromide (CTAB) method introduced by Doyle and Doyle [26]. The concentration and quality of DNA was checked by Nanodrop spectrophotometer (Thermo Fisher Scientific, Waltham, MA, USA). To enrich the viral load, rolling circle amplification (RCA) was carried by phi 29 DNA polymerase (Thermo Fisher Scientific) using 200 ng/µL genomic DNA as template and following the standard protocol [27].

### Cloning of begomoviruses and associated satellites and their Sanger sequencing

Samples from seven major districts of Punjab (Vehari, Multan, Khanewal, Toba Tek Singh, Burewala, Sahiwal, and Faisalabad) were selected for cloning and sequencing of begomoviruses and associated DNA satellites. The genomic DNA was used as template in PCR with begomoviruses universal primers [28], betasatellites universal primers [29], and alphasatellites universal primers [30]. The PCR products were cloned in T/A cloning vector, i.e. pTZ57R/T (Thermo Fisher Scientific). Three clones of begomoviruses from each district and multiple clones of satellites were Sanger sequenced in both orientations employing a primer walking strategy using Applied Biosystems 3730XL DNA sequencer (USA).

### NGS of RCA products

RCA products of five samples from different locations were pooled and used for NGS with a recently introduced method: CIDER-Seq (Circular DNA Enrichment sequencing) for the unbiased enrichment and long-read sequencing of viral-sized circular DNA molecules. We followed this method as described in Mehta *et al.* [25] with some modifications (for downstream analysis of the data generated). Full-length reads of each molecule were filtered with >99% quality and they were also de-cancatenated using the similar algorithm used earlier [25]. Finally, we obtained 9829 sequenced molecules (“reads”). The next challenge was to identify each molecule. For this purpose, we prepared some scripts in python to obtain the accurate and fast results without any human error.

### BLASTn analysis of each sequence (read) and downstream screening/selection

First, we did BLASTn (Basic Local Alignment Searching Tool for nucleotides) of each sequence to obtain the top 10 [10] similar targets. The similarity data were downloaded in excel file and prepared the final file containing the targets of all the 9829 sequences. Then we filtered the most targets of each sequence and based on the same target, sequences were separated into the groups and gave the putative name to each group like CLCuMuV-Raj (includes all those sequences whose target was this virus in BLASTn). Finally, we assigned all sequences into different groups and separated the groups with viruses and associated satellites molecules.

### Designing of scripts in python to solve technical issues in data analysis

The length of these sequences in each group was in the range starting from few hundred nucleotides to the full-length size of each virus or satellite molecule so it was important to separate them based on size. For this we designed a script in which we just provided the input fasta file and a reference file consisting of the name of each sequence. Ultimately, it gives two output files, one consisting of the selected fasta file and the second is with the names of sequences in one column and the size of the sequences in next column. So, this can be copied in the excel and filtered for the sequences of the desired sizes. Second issue was to separate the sequences with conserved nonancleotide (TAATATTAC for begomoviruses/betasatellites and TAGTATTAC for alphasatellites). That was carried out in excel and the next issue was that some sequences had this conserved nonanucleotide sequences in the complementary sequences so we first determined their reverse-complement online using http://www.bioinformatics.org/sms2/rev_comp.html platform. After that all sequences were in virion strand but the position of nonanucleotide was at different positions in each sequence. So, for further downstream analysis it was necessary to start each sequence with AC of nonanucleotide and end its TAATATT and similarly for alphasatellites it should end with TAGTATT. For this we designed a script that takes a fasta input file and generates the output file with desired results. As manually it was very time taking and there were chances of human errors.

### Sanger sequenced reads assembly, BLASTn analysis, and ORFs findings

Sequenced reads were assembled in SeqMan (Lasergene, DNAStar Inc., Madison, WI, USA) and after trimming the vector portion, a consensus contig was saved and analyzed. Complete sequence of both begomoviruses and satellites was searched in NCBI database by BLASTn using default settings of BLASTn to get the most similar sequences. The open-reading frames (ORFs) were found using the NCBI online tool for ORFs findings (https://www.ncbi.nlm.nih.gov/orffinder/).

### Sequence demarcation tool analysis for species and strain demarcation

For species and strain demarcation, sequence demarcation tool (SDT) program [31] was used following the taxonomic criteria of begomoviruses, i.e. 91% and 94% pairwise sequence identity (PSI) for species and strain, respectively [32] and of alphasatellites 88% and 70% PSI for species and genus demarcation [33]. For betasatellites, previously recommended species demarcation was 78% PSI scores [34] whereas recently there is a proposal list by Bridden *et al*., on ICTV with 91% PSI scores for species demarcation of betasatellite and no cutoff for strain demarcation. A MUSCLE alignment of these newly isolated begomoviruses with their top 100 most similar sequences (retrieved by BLASTn of these new isolates on NCBI database) and other related begomoviruses available on ICTV was performed using default settings of SDT program. Similarly, PSI scores of satellites were also determined.

### Phylogenetic analysis of complete sequences of begomoviruses and satellites

The multiple sequence alignment (MSA) was performed by MUSCLE program [35] in MEGA7 [36] software. Using Neighbor-joining method [37] with bootstrapping test [38] in MEGA7, a phylogenetic tree was reconstructed to infer the relatedness of these newly identified virus sequences with the other reported begomoviruses. Similarly, sequences of both betasatellite and alphasatellites were phylogenetically analyzed for their relationship with other already reported satellites.

### Detection of possible recombination events in viral and satellite molecules

Sequences of multiple begomoviruses reported from the Indian sub-continent since 1990s were retrieved from NCBI database. MSA was carried out by MUSCLE in MEGA7. For the detection of recombination events, we used the latest version of Recombination Detection Program (RDP), i.e. RDP4 [39]. RDP using nine recombination analysis methods, RDP [40], GENECONV [41], BOOTSCAN [42], MAXIMUM CHI SQUARE [43], CHIMAERA [44], SISCAN [45], 3SEQ [46], PHYLPRO [47], LARD [48], and VisRD [49] for possible recombination events within query sequences.

### Detection of specific mutations in genes and intergenic region of the distinct strain of CLCuMuV and betasatellite

As we isolated the single distinct strain of monopartite begomovirus, i.e. CLCuMuV-Raj in this epidemic of CLCuD, it was important to know the changes at protein level in this strain which distinguishes it from others. For this purpose, we retrieved the sequences of all the begomoviruses reported in the Indian sub-continent since 1990s and after generating a phylogenetic tree we identified CLCuMuV species group. We retrieved the protein sequences of all the isolates of the CLCuMuV in the group. MSA of protein sequences was carried out by MUSCLE program [35] in MEGA7 [36] software. We were able to find out the novel mutations at specific positions in different genes of recently identified isolates of begomoviruses associated with this epidemic in Pakistan as well as associated with previously identified CLCuMuV strains in India and Pakistan in 2015 and afterward.

### Quantitative real-time PCR for relative levels of viruses and associated satellites

The genomic DNA extracted from three infected samples (from three different locations) was used as a template for quantitative real-time PCR (qRT-PCR) to determine the relative titer of CLCuMuV-Raj and associated satellites identified in current study.

### Screening of samples using CP primers of CLCuMuV-Raj strain amplification and sequencing

Primers (CLCuMuV-Raj-CP-F 5′-ATGTCGAAGCGAGCTGCCGA-3′ and CLCuMuV-Raj-CP-R 5′-TCAATTCGTTACAGAGTCAT-3′) were designed to amplify the CP of CLCuMuV-Raj strain and all the symptomatic samples were screened for the detection of this virus spread. Forty PCR products from different locations were sequenced.

## Results

### Pacbio data analysis

**Figure 3:**
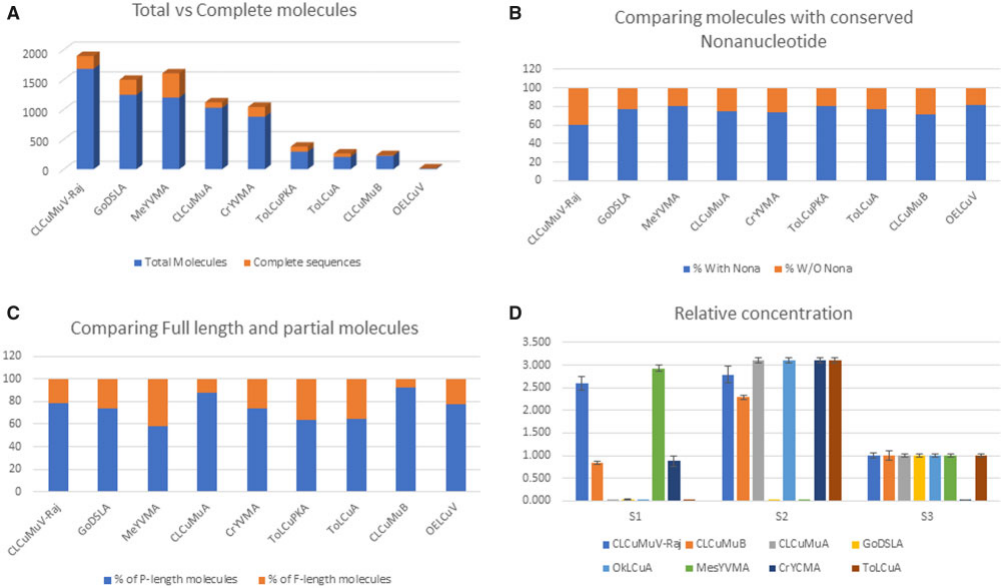
(**A**) The total number of molecules of each species versus the total number of complete sequences of each species of virus and satellites identified in the Pacbio data analysis. (**B**) Compared the molecules of each species with the presence and absence of conserved nonanucleotides sequence. (**C**) Compared the length of molecules that have conserved nonanucleotides sequence. (**D**) Relative titer of each species in three independent samples (S1–S3).

After BLASTn of each read we divided all the reads into groups according to their top hits in BLASTn analysis and downstream analysis for their species and strain demarcation. In Fig. 3(A–C), we summarized the results of pacbio data analysis. Figure 3(A) shows the total number of molecules of each species versus the total number of complete sequences of each species of virus and satellites identified in the Pacbio data analysis. Figure 3(B) shows the molecules of each species with the presence and absence of conserved nonanucleotides [TAATATTAC (for begomoviruse and betasatellite) and TAGTATTAC for alphasatellites] sequence. Figure 3(C) shows the percentage of the full-length molecules that have conserved nonanucleotides sequence.

### BLASTn and ORFs findings of complete virus and satellite sequences

The BLASTn results of all the sequences of begomoviruses showed that they were ≥98% similar to six isolates of CLCuMuV (MG373556, MG373551, KY120359-61, and MF184923) isolated from India recently in three different studies and three isolates (KX656806 and KX656809-10) identified in 2015 from Vehari, Pakistan. BLASTn results of all betasatellites sequences showed ≥98% sequence identity to different isolates of CLCuMuB identified from cotton and *B. tabaci* in the last 4–5 years, both from India and Pakistan. BLASTn of alphasatellites sequences from 16 clones showed maximum similarity of 97% to one isolate of Cotton leaf curl Multan alphasatellite (CLCuMuA) (MF344548) and 94% with few other isolates of CLCuMuA isolated from cotton in the Indian sub-continent. Interestingly, they showed 97% sequence identity with just 78% query coverage with all other isolates of CLCuMuA. Eleven sequences of alphasatellite showed ≥94% few isolates of Tomato leaf curl alphasatellite (ToLCA) identified from both countries in the last few years. All other 18 sequences of alphasatellite showed ≥94% sequence identity with few isolates of okra leaf curl alphasatellite isolated from different hosts in the last few years in both countries. BLASTn results of both satellites indicate that they have recently evolved with their helper viruses in the disease complex. The ORFs of all the isolates of CLCuMuV and satellites are shown in Tables 1 and 2, respectively.

**Table 1:** reading frames of CLCuMuV

Sr. No	Accession Numbers	Virus name	Isolate/clone	Area of isolation	Virus component size (bp)	C1	C2	C3	(A)C4	C5	V1	V2
						Start-end (product size aa)	Start-end (product size aa)	Start-end (product size aa)	Start-end (product size aa)	Start-end (product size aa)	Start-end (product size aa)	Start-end (product size aa)
1	MK357244	CLCuMuV	NAS-1	Vehari	2738	2583–1495 (362)	1598–1146 (150)	1453–1049 (134)	2429–2127 (100)	791–60 (243)	276–1046 (256)	116–472 (118)
2	MK357245	CLCuMuV	NAS-2	Vehari	2738	2583–1495 (362)	1598–1146 (150)	1453–1049 (134)	2429–2127 (100)	791–60 (243)	276–1046 (256)	116–472 (118)
3	MK357246	CLCuMuV	NAS-3	Vehari	2738	2583–1495 (362)	1598–1146 (150)	1453–1049 (134)	2429–2127 (100)	791–60 (243)	276–1046 (256)	116–472 (118)
4	MK357247	CLCuMuV	NAS-4	Burewala	2738	2583–1495 (362)	1598–1146 (150)	1453–1049 (134)	2429–2127 (100)	791–60 (243)	276–1046 (256)	116–472 (118)
5	MK357248	CLCuMuV	NAS-5	Burewala	2738	2583–1495 (362)	1598–1146 (150)	1453–1049 (134)	2429–2127 (100)	791–60 (243)	276–1046 (256)	116–472 (118)
6	MK357249	CLCuMuV	NAS-6	Burewala	2738	2583–1495 (362)	1598–1146 (150)	1453–1049 (134)	2429–2127 (100)	791–60 (243)	276–1046 (256)	116–472 (118)
7	MK357250	CLCuMuV	NAS-7	Multan	2738	2583–1495 (362)	1598–1146 (150)	1453–1049 (134)	2429–2127 (100)	791–60 (243)	276–1046 (256)	116–472 (118)
8	MK357251	CLCuMuV	NAS-8	Multan	2738	2583–1495 (362)	1598–1146 (150)	1453–1049 (134)	2429–2127 (100)	791–60 (243)	276–1046 (256)	116–472 (118)
9	MK357252	CLCuMuV	NAS-9	Multan	2738	2583–1495 (362)	1598–1146 (150)	1453–1049 (134)	2429–2127 (100)	791–60 (243)	276–1046 (256)	116–472 (118)
10	MK357253	CLCuMuV	NAS-10	Sahiwal	2738	2583–1495 (362)	1598–1146 (150)	1453–1049 (134)	2429–2127 (100)	791–60 (243)	276–1046 (256)	116–472 (118)
11	MK357254	CLCuMuV	NAS-11	Sahiwal	2738	2583–1495 (362)	1598–1146 (150)	1453–1049 (134)	2429–2127 (100)	791–60 (243)	276–1046 (256)	116–472 (118)
12	MK357255	CLCuMuV	NAS-12	Sahiwal	2738	2583–1495 (362)	1598–1146 (150)	1453–1049 (134)	2429–2127 (100)	791–60 (243)	276–1046 (256)	116–472 (118)
13	MK357256	CLCuMuV	NAS-13	Faisalabad	2738	2583–1495 (362)	1598–1146 (150)	1453–1049 (134)	2429–2127 (100)	791–60 (243)	276–1046 (256)	116–472 (118)
14	MK357257	CLCuMuV	NAS-14	Faisalabad	2738	2583–1495 (362)	1598–1146 (150)	1453–1049 (134)	2429–2127 (100)	791–60 (243)	276–1046 (256)	116–472 (118)
15	MK357258	CLCuMuV	NAS-15	Faisalabad	2738	2583–1495 (362)	1598–1146 (150)	1453–1049 (134)	2429–2127 (100)	791–60 (243)	276–1046 (256)	116–472 (118)
16	MT037028	CLCuMuV	NAS-85	Khanewal	2738	2583–1495 (362)	1598–1146 (150)	1453–1049 (134)	2429–2127 (100)	791–60 (243)	276–1046 (256)	116–472 (118)
17	MT037029	CLCuMuV	NAS-86	Khanewal	2738	2583–1495 (362)	1598–1146 (150)	1453–1049 (134)	2429–2127 (100)	791–60 (243)	276–1046 (256)	116–472 (118)
18	MT037030	CLCuMuV	NAS-87	Khanewal	2738	2583–1495 (362)	1598–1146 (150)	1453–1049 (134)	2429–2127 (100)	791–60 (243)	276–1046 (256)	116–472 (118)
19	MT037031	CLCuMuV	NAS-88	Toba Tek Singh	2738	2583–1495 (362)	1598–1146 (150)	1453–1049 (134)	2429–2127 (100)	791–60 (243)	276–1046 (256)	116–472 (118)
20	MT037032	CLCuMuV	NAS-89	Toba Tek Singh	2738	2583–1495 (362)	1598–1146 (150)	1453–1049 (134)	2429–2127 (100)	791–60 (243)	276–1046 (256)	116–472 (118)
21	MT037033	CLCuMuV	NAS-90	Toba Tek Singh	2738	2583–1495 (362)	1598–1146 (150)	1453–1049 (134)	2429–2127 (100)	791–60 (243)	276–1046 (256)	116–472 (118)
22	MT037052	dCLCuMuV	NAS-109	Vehari	1363	1208–60 (382)^**^	–	–	1054–752 (100)	–	276–515 (79)^*^	116–472 (118)
23	MT037053	dCLCuMuV	NAS-110	Vehari	1363	1208–60 (382)^**^	–	–	1054–752 (100)	–	276–515 (79)^*^	116–472 (118)
24	MT037054	dCLCuMuV	NAS-111	Khanewal	1363	1208–60 (382)^**^	–	–	1054–752 (100)	–	276–515 (79)^*^	116–472 (118)
25	MT037055	dCLCuMuV	NAS-112	Khanewal	1363	1208–60 (382)^**^	–	–	1054–752 (100)	–	276–515 (79)^*^	116–472 (118)
26	MT037056	dCLCuMuV	NAS-113	Toba Tek Singh	1363	1208–60 (382)^**^	–	–	1054–752 (100)	–	276–515 (79)^*^	116–472 (118)
27	MT037057	dCLCuMuV	NAS-114	Toba Tek Singh	1363	1208–60 (382)^**^	–	–	1054–752 (100)	–	276–515 (79)^*^	116–472 (118)
28	MT037058	dCLCuMuV	NAS-115	Faisalabad	1413	1258–497 (253)^*^	–	–	1104–802 (100)	–	276–512 (78)^*^	116–472 (118)
29	MT037059	dCLCuMuV	NAS-116	Multan	1413	1258–497 (253)^*^	–	–	1104–802 (100)	–	276–512 (78)^*^	116–472 (118)
30	MT037060	dCLCuMuV	NAS-117	Sahiwal	1413	1258–497 (253)^*^	–	–	1104–802 (100)	–	276–512 (78)^*^	116–472 (118)

The product size of each gene is also shown in number of amino acids (aa) it codes for. The Genebank accession number assigned to each virus in current study, its clone, isolation area, and genome size (bp) is also mentioned in respective column of each isolate.

* Partial sequence.

** Larger size.

**Table 2:** reading frames of all isolates of betasatellites and alphasatellites identified in current study are shown

Sr. No	Accession numbers	Name of satellites	Isolate/clone	Area of isolation	Size of the satellites	βC1	Rep
						Start-end (product size aa)	Start-end (product size aa)
1	MK357271	CLCuMuB	NAS-28	Multan	1358	550–194 (118)	−
2	MK357272	CLCuMuB	NAS-29	Multan	1358	550–194 (118)	−
3	MK357273	CLCuMuB	NAS-30	Multan	1358	550–194 (118)	−
4	MK357274	CLCuMuB	NAS-31	Burewala	1358	550–194 (118)	−
5	MK357275	CLCuMuB	NAS-32	Burewala	1358	550–194 (118)	−
6	MK357276	CLCuMuB	NAS-33	Burewala	1358	550–194 (118)	−
7	MK357277	CLCuMuB	NAS-34	Vehari	1358	550–194 (118)	−
8	MK357278	CLCuMuB	NAS-35	Vehari	1358	550–194 (118)	−
9	MK357279	CLCuMuB	NAS-36	Vehari	1358	550–194 (118)	−
10	MK357280	CLCuMuB	NAS-37	Faisalabad	1358	550–194 (118)	−
11	MK357281	CLCuMuB	NAS-38	Faisalabad	1358	550–194 (118)	−
12	MK357282	CLCuMuB	NAS-39	Faisalabad	1358	550–194 (118)	−
13	MK357283	CLCuMuB	NAS-40	Sahiwal	1358	550–194 (118)	−
14	MK357284	CLCuMuB	NAS-41	Sahiwal	1358	550–194 (118)	−
15	MK357285	CLCuMuB	NAS-42	Sahiwal	1358	550–194 (118)	−
16	MT037034	CLCuMuB	NAS-91	Khanewal	1358	550–194 (118)	−
17	MT037035	CLCuMuB	NAS-92	Khanewal	1358	550–194 (118)	−
18	MT037036	CLCuMuB	NAS-93	Khanewal	1358	550–194 (118)	−
19	MT037037	CLCuMuB	NAS-94	Khanewal	1358	550–194 (118)	−
20	MT037038	CLCuMuB	NAS-95	Toba Tek Singh	1358	550–194 (118)	−
21	MT037039	CLCuMuB	NAS-96	Toba Tek Singh	1358	550–194 (118)	−
22	MT037040	CLCuMuB	NAS-97	Toba Tek Singh	1358	550–194 (118)	−
23	MT037041	CLCuMuB	NAS-98	Toba Tek Singh	1358	550–194 (118)	−
24	MT037042	CLCuMuB	NAS-99	Toba Tek Singh	1358	550–194 (118)	−
25	MK357286	CLCuMuA	NAS-43	Vehari	1370	−	77–1024 (315)
26	MK357287	CLCuMuA	NAS-44	Multan	1379	−	77–1024 (315)
27	MK357288	CLCuMuA	NAS-45	Multan	1378	−	77–1024 (315)
28	MK357289	CLCuMuA	NAS-46	Multan	1381	−	77–1024 (315)
29	MK357290	CLCuMuA	NAS-47	Multan	1380	−	77–1024 (315)
30	MK357291	CLCuMuA	NAS-48	Burewala	1380	−	77–1024 (315)
31	MK357292	CLCuMuA	NAS-49	Burewala	1379	−	77–1024 (315)
32	MK357293	CLCuMuA	NAS-50	Burewala	1379	−	77–1024 (315)
33	MK357294	CLCuMuA	NAS-51	Sahiwal	1378	−	77–1024 (315)
34	MK357295	CLCuMuA	NAS-52	Sahiwal	1379	−	77–1024 (315)
35	MK357296	CLCuMuA	NAS-53	Sahiwal	1378	−	77–1024 (315)
36	MK357297	CLCuMuA	NAS-54	Faisalabad	1380	−	77–1024 (315)
37	MK357298	CLCuMuA	NAS-55	Faisalabad	1378	−	77–1024 (315)
38	MK357299	ToLCuA	NAS-56	Vehari	1371	−	74–1021 (315)
39	MK357300	ToLCuA	NAS-57	Vehari	1371	−	74–1021 (315)
40	MK357301	ToLCuA	NAS-58	Vehari	1371	−	74–1021 (315)
41	MK357302	ToLCuA	NAS-59	Burewala	1371	−	74–1021 (315)
42	MK357303	ToLCuA	NAS-60	Burewala	1371	−	74–1021 (315)
43	MK357304	ToLCuA	NAS-61	Multan	1371	−	74–1021 (315)
44	MK357305	ToLCuA	NAS-62	Multan	1371	−	74–1021 (315)
45	MK357306	ToLCuA	NAS-63	Sahiwal	1371	−	74–1021 (315)
46	MK357307	ToLCuA	NAS-64	Sahiwal	1371	−	74–1021 (315)
47	MK357308	ToLCuA	NAS-65	Faisalabad	1371	−	74–1021 (315)
48	MK357309	ToLCuA	NAS-66	Faisalabad	1371	−	74–1021 (315)
49	MK357310	OkLCuA	NAS-67	Vehari	1373	−	82–1029 (315)
50	MK357311	OkLCuA	NAS-68	Vehari	1373	−	82–1029 (315)
51	MK357312	OkLCuA	NAS-69	Vehari	1373	−	82–1029 (315)
52	MK357313	OkLCuA	NAS-70	Burewala	1373	−	82–1029 (315)
53	MK357314	OkLCuA	NAS-71	Burewala	1373	−	82–1029 (315)
54	MK357315	OkLCuA	NAS-72	Faisalabad	1373	−	82–1029 (315)
55	MK357316	OkLCuA	NAS-73	Faisalabad	1373	−	82–1029 (315)
56	MK357317	OkLCuA	NAS-74	Sahiwal	1373	−	82–1029 (315)
57	MK357318	OkLCuA	NAS-75	Sahiwal	1373	−	82–1029 (315)
58	MK357319	OkLCuA	NAS-76	Multan	1373	−	82–1029 (315)
59	MK357320	OkLCuA	NAS-77	Multan	1373	−	82–1029 (315)
60	MK357321	OkLCuA	NAS-78	Multan	1373	−	82–1029 (315)
61	MK357322	OkLCuA	NAS-79	Vehari	1365	−	82–1029 (315)
62	MK357323	OkLCuA	NAS-80	Burewala	1366	−	82–1029 (315)
63	MK357324	OkLCuA	NAS-81	Burewala	1365	−	82–1029 (315)
64	MK357325	OkLCuA	NAS-82	Multan	1364	−	82–1029 (315)
65	MK357326	OkLCuA	NAS-83	Multan	1368	−	82–1029 (315)
66	MK357327	OkLCuA	NAS-84	Faisalabad	1375	−	82–1029 (315)
67	MT037043	GoDSLA	NAS-100	Khanewal	1372	−	70–1017 (315)
68	MT037044	GoDSLA	NAS-101	Khanewal	1372	−	70–1017 (315)
69	MT037045	GoDSLA	NAS-102	Toba Tek Singh	1372	−	70–1017 (315)
70	MT037046	CLCuMuA	NAS-103	Khanewal	1371	−	77–1024 (315)
71	MT037047	CLCuMuA	NAS-104	Toba Tek Singh	1371	−	77–1024 (315)
72	MT037048	CLCuMuA	NAS-105	Toba Tek Singh	1371	−	77–1024 (315)
73	MT037049	OkLCuA	NAS-106	Toba Tek Singh	1373	−	82–1029 (315)
74	MT037050	OkLCuA	NAS-107	Toba Tek Singh	1373	−	82–1029 (315)
75	MT037051	OkLCuA	NAS-108	Khanewal	1373	−	82–1029 (315)
76	MT037061	dCLCuMuB	NAS-118	Multan	776	−	−
77	MT037062	dCLCuMuB	NAS-119	Vehari	651	−	−
78	MT037063	dCLCuMuB	NAS-120	Khanewal	684	−	−

The product size of each gene is also shown in number of amino acids (aa) it codes for. The Genebank accession number assigned to each sequence, its clone name, isolation area, and genome size (bp) is also mentioned in respective column of each isolate.

### Species and strain demarcation

SDT analysis showed that our newly isolated 15 clones of CLCuMuV have more than 99% sequence identity among themselves and they also showed more than 98% PSI with three isolates of CLCuMuV (KY120359-61) identified from Barnala, district of Punjab, India in 2015 [23] and one sequence (MF184923; identified on cotton and sequence submitted in 2017 in NCBI) and 99% PSI with two isolates of CLCuMuV identified from hollyhock in New Delhi, India in 2016. They also showed more than 98% sequence identity with three isolates of CLCuMuV (KX656806 and KX656809-10) identified from Vehari district of Punjab, Pakistan in 2015 [21]. SDT analysis for strains and species using representative isolates of each strain showed that all current isolates have 94–95% PSI with CLCuMuV-Raj strain and CLCuMuV-PK strain of CLCuMuV. SDT analysis of all sequences of betasatellites showed more than 99% sequence identity among all clones and 99% identity with three CLCuMuB isolates (KY523521, KT228323, and MF141730 host; cotton) from India, one isolate (LT608338; host; *Luffa cylindrica*), three isolates (KX656830, KX656835, and KX697597 host; cotton) from Pakistan. Using the current criteria of species and genus demarcation cutoff values 88% and 70%, respectively, for alphasatellites we identified seven species of alphasatellites; *Cotton leaf curl Multan alphasatellite* (CLCuMuA), ToLCA, *Tomato leaf curl Pakistan alphasatellite* (ToLCPKA), *Gossypium darwinii symptomless alphasatellite* (GoDarSLA), *Okra leaf curl alphasatellite* (OkLCA), *Mesta yellow vein mosaic alphasatellite* (MeYVMA), and *Croton yellow vein mosaic alphasatellite* (CrYVMA) in three different genera.

### Phylogenetic tree analysis

**Figure 4:**
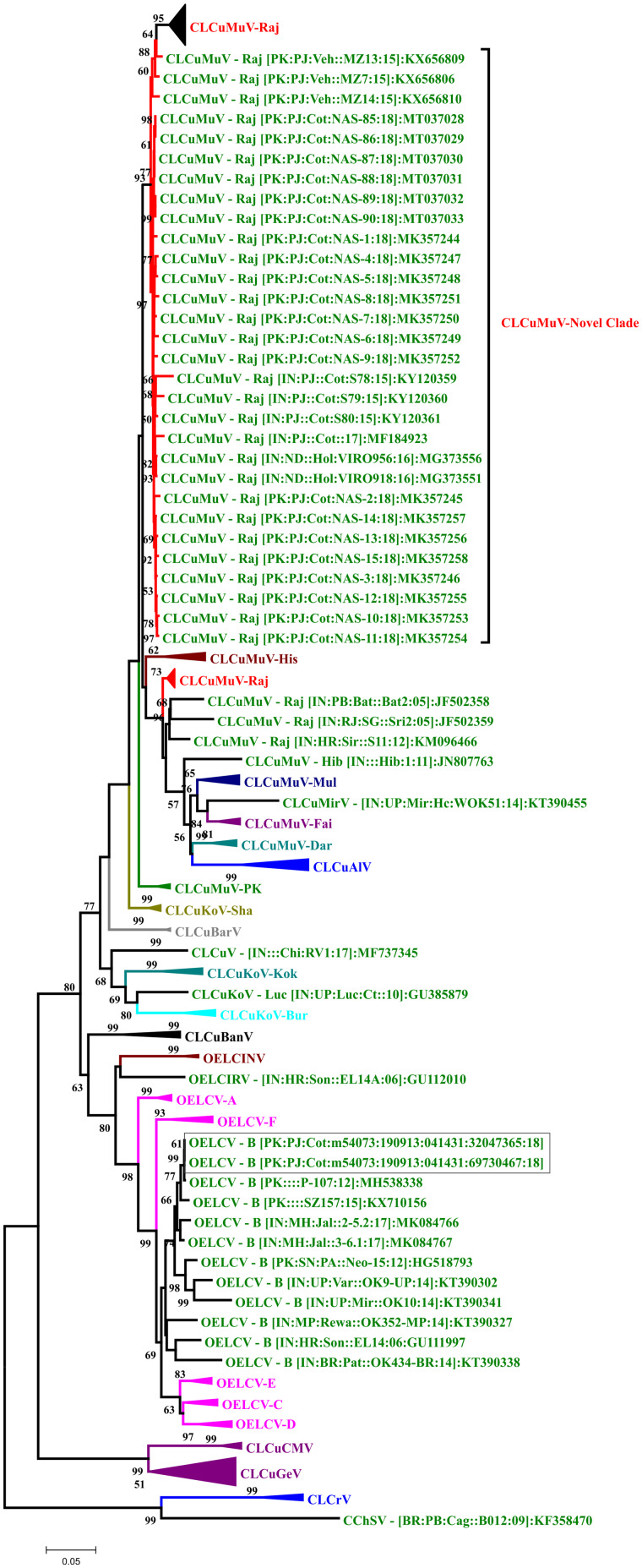
neighbor-joining phylogenetic tree was reconstructed using the full-length genome sequences of different species of cotton begomoviruses. All the isolates of CLCuMuV identified in current study developed a novel clade in the major clade of CLCuMuV-Raj strain. We propose this novel clade associated with third epidemic of CLCuD in Indian sub-continent. Neighbor-joining method was used to reconstruct the tree using bootstrap test with 1000 replicates (percentage boopstrap value is shown on each branch). Two complete isolates of OELCV identified in current study are shown in square brackets. Species abbreviations used in this tree are following: *Cotton leaf curl Khokran virus* (CLCuKoV), *Cotton leaf curl Alabad virus* (CLCuAlV), *Cotton leaf curl Bangalore virus* (CLCuBanV), *Cotton leaf curl Cameroon virus* (CLCuCMV), *Cotton leaf curl Barasat virus* (CLCuBarV), *Okra enation leaf curl virus* (OELCV), *Cotton leaf curl Gezira virus* (CLCuGeV), *Cotton leaf crumple virus* (CLCrV), and *Cotton chlorotic spot virus* (CChSV).

**Figure 5:**
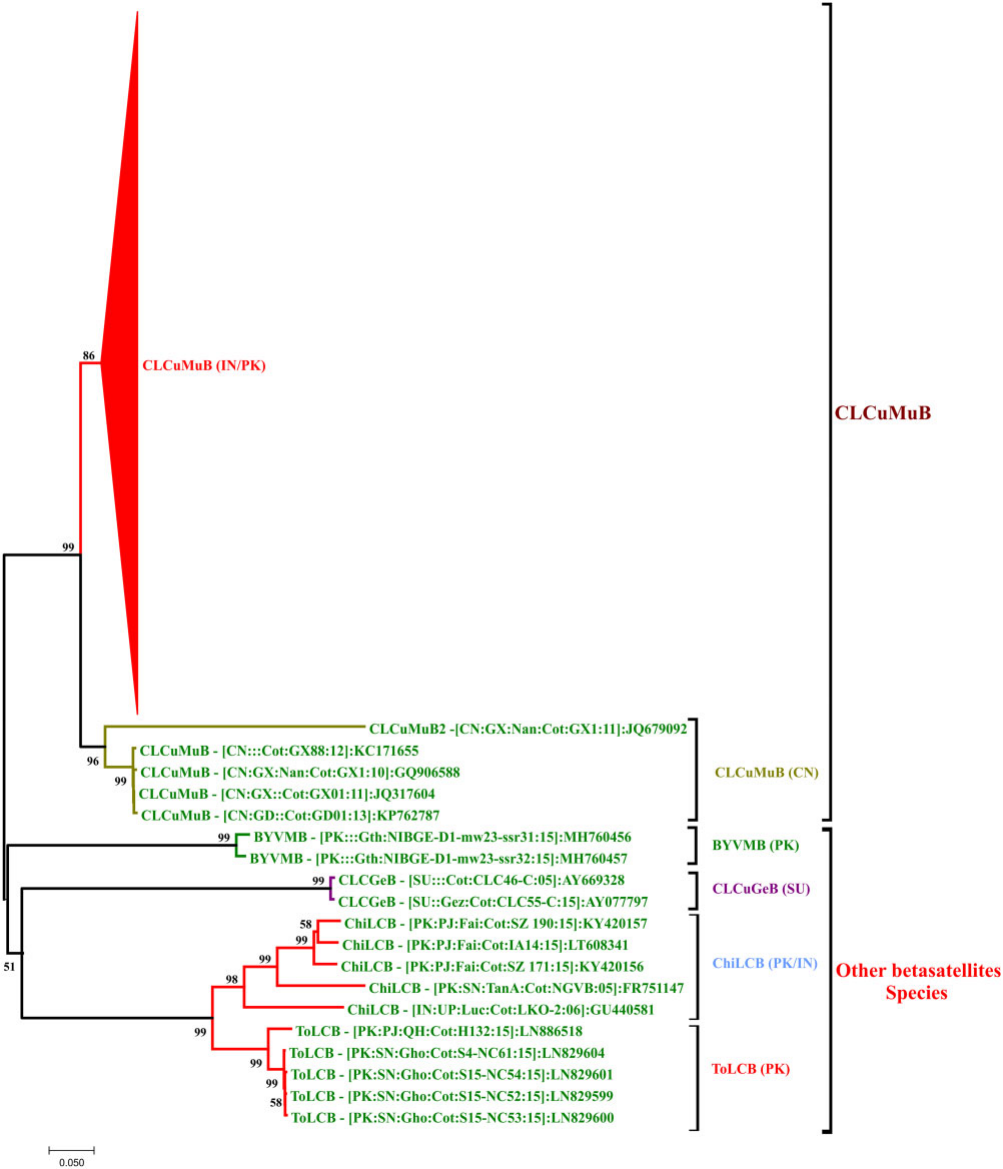
neighbor-joining phylogenetic tree of CLCuMuB constructed using the full-length genome sequences of all isolates of betasatellites so for identified from cotton species and the most identical sequences of betasatellites to the isolates of current study. Neighbor-joining method was used with bootstrap test (1000 replicates and percentage bootstrap value is shown on each branch). All isolates of current study were present in the main clade of CLCuMuB (identified from cotton in the Indian sub-continent). Isolates of CLCuMuB reported in cotton from China made a separate clade where one isolate is quite different and below the cutoff values of betasatellites to which we called CLCuMuB2 here. Other species of betasatellites so far reported in cotton are *Bhendi yellow vein mosaic betasatellite* (BYVMB), *Chili leaf curl betasatellite* (ChiLCB), and *Tomato leaf curl betasatellite* (ToLCB).

**Figure 6:**
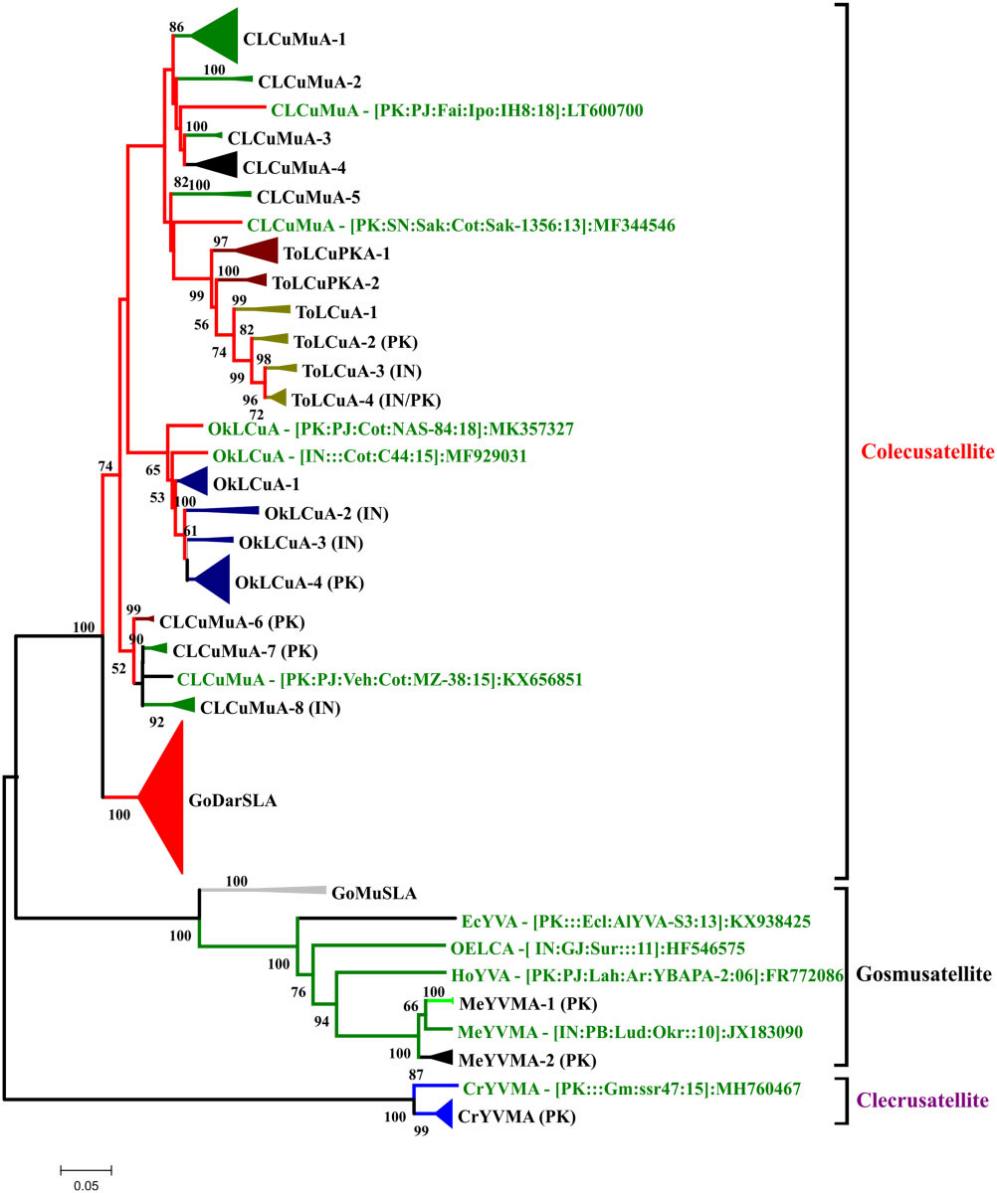
neighbor-joining phylogenetic tree of all alphasatellites isolated in current study was reconstructed to infer their relationship with already reported isolates of alphasatellites. Bootstrap test was applied with 1000 replicates to validate the significant confidence level of each branch and its values in percentage are shown on each branch. In current study, we identified seven species of alphasatellites: *Cotton leaf curl Multan alphasatellite* (CLCuMuA), *Tomato leaf curl Pakistan alphasatellite* (ToLCPKA), *Gossypium darwinii symptomless alphasatellite* (GoDarSLA), *Okra leaf curl alphasatellite* (OkLCA), and *Croton yellow vein mosaic alphasatellite* (CrYVMA). Other species of alphasatellites used in this tree are *Gossypium mustilinum symptomless alphasatellite* (GoMuSLA), *Eclipta yellow vein alphasatellite* (EcYVA), *Hollyhock yellow vein alphasatellite* (HoYVA), and *Okra enation leaf curl alphasatellite* (OELCA).

A neighbor-joining phylogenetic tree of all isolates of CLCuMuV made a novel clade between the isolates of CLCuMuV-Raj strain. This novel clade also includes recently identified nine isolates (three from Pakistan and six from India). Two isolates of OELCV identified in the current study were present in the clade of OELCV with its recent isolates from whitefly and cotton in Pakistan. OELCV also show great genetic diversity and we numbered different strains by A–G and our isolates were present within B strain (Fig. 4). Phylogenetic tree constructed using all isolates of betasatellites identified in current study showed that they all were present in the clade of CLCuMuB identified recently from cotton in the Indian subcontinent (Fig. 5). Phylogenetic tree using all sequences of the alphasatellites isolated in the current study and all the isolates of the same species of each isolate represents that there are seven species of alphasatellites identified in the current study (Fig. 6).

### Detection of recombination events in CLCuMuV

The possible recombination events in the isolates of novel clade of CLCuMuV were determined by RDP4 [39]. A recombination event with significant *P*-values was found in all recent isolates of CLCuMuV. In this event, major parent was CLCuMuV-PK (EU365616) and minor parent was CLCuKoV-Bur (JF502353). RDP analysis results are shown in Table 3.

**Table 3:** recombination events detected in all isolates of distinct strain of CLCuMuV identified in current study and previously identified from both India and Pakistan since 2015

Accession No. of recombinant Of CLCuMuV	Recombination breakpoints		Parent-like sequences		Methods that detected recombination						
					Average *p*-value						
	Beginning breakpoint	Ending breakpoint	Major (Acronym, Accession # & %age similarity	Minor (Acronym, Accession # & %age similarity)	RDP	GENECONV	BootScan	MaxChi	Chimaera	SiScan	3Seq
MK357244	379	887	CLCuMuV-PK EU365616 (98.9%)	CLCuKoV-Bur (JF502353) (99.8%)	2.998 × 10^−60^	4.847 × 10^−60^	2.748 × 10^−41^	1.113 × 10^−19^	6.866 × 10^−20^	1.271 × 10^−23^	1.067 × 10^−10^
MK357245	379	887	CLCuMuV-PK EU365616 (98.5%)	CLCuKoV-Bur (JF502353) (99.6%)	2.998 × 10^−60^	4.847 × 10^−60^	2.748 × 10^−41^	1.113 × 10^−19^	6.866 × 10^−20^	1.271 × 10^−23^	1.067 × 10^−10^
MK357246	379	887	CLCuMuV-PK EU365616 (98.9%)	CLCuKoV-Bur (JF502353) (99.8%)	2.998 × 10^−60^	4.847 × 10^−60^	2.748 × 10^−41^	1.113 × 10^−19^	6.866 × 10^−20^	1.271 × 10^−23^	1.067 × 10^−10^
MK357247	328	887	CLCuMuV-PK EU365616 (99%)	CLCuKoV-Bur (JF502353) (99.5%)	2.998 × 10^−60^	4.847 × 10^−60^	2.748 × 10^−41^	1.113 × 10^−19^	6.866 × 10^−20^	1.271 × 10^−23^	1.067 × 10^−10^
MK357248	376	887	CLCuMuV-PK EU365616 (98.8%)	CLCuKoV-Bur (JF502353) (99.4%)	2.998 × 10^−60^	4.847 × 10^−60^	2.748 × 10^−41^	1.113 × 10^−19^	6.866 × 10^−20^	1.271 × 10^−23^	1.067 × 10^−10^
MK357249	342	887	CLCuMuV-PK EU365616 (99%)	CLCuKoV-Bur (JF502353) (99.1%)	2.998 × 10^−60^	4.847 × 10^−60^	2.748 × 10^−41^	1.113 × 10^−19^	6.866 × 10^−20^	1.271 × 10^−23^	1.067 × 10^−10^
MK357250	395	887	CLCuMuV-PK EU365616 (98.9%)	CLCuKoV-Bur (JF502353) (99.6%)	2.998 × 10^−60^	4.847 × 10^−60^	2.748 × 10^−41^	1.113 × 10^−19^	6.866 × 10^−20^	1.271 × 10^−23^	1.067 × 10^−10^
MK357251	395	887	CLCuMuV-PK EU365616 (98.7%)	CLCuKoV-Bur (JF502353) (99.4%)	2.998 × 10^−60^	4.847 × 10^−60^	2.748 × 10^−41^	1.113 × 10^−19^	6.866 × 10^−20^	1.271 × 10^−23^	1.067 × 10^−10^
MK357252	376	887	CLCuMuV-PK EU365616 (99%)	CLCuKoV-Bur (JF502353) (99%)	2.998 × 10^−60^	4.847 × 10^−60^	2.748 × 10^−41^	1.113 × 10^−19^	6.866 × 10^−20^	1.271 × 10^−23^	1.067 × 10^−10^
MK357253	379	887	CLCuMuV-PK EU365616 (98.7%)	CLCuKoV-Bur (JF502353) (99.8%)	2.998 × 10^−60^	4.847 × 10^−60^	2.748 × 10^−41^	1.113 × 10^−19^	6.866 × 10^−20^	1.271 × 10^−23^	1.067 × 10^−10^
MK357254	379	887	CLCuMuV-PK EU365616 (98.8%)	CLCuKoV-Bur (JF502353) (99.6%)	2.998 × 10^−60^	4.847 × 10^−60^	2.748 × 10^−41^	1.113 × 10^−19^	6.866 × 10^−20^	1.271 × 10^−23^	1.067 × 10^−10^
MK357255	376	887	CLCuMuV-PK EU365616 (98.9%)	CLCuKoV-Bur (JF502353) (99.8%)	2.998 × 10^−60^	4.847 × 10^−60^	2.748 × 10^−41^	1.113 × 10^−19^	6.866 × 10^−20^	1.271 × 10^−23^	1.067 × 10^−10^
MK357256	328	887	CLCuMuV-PK EU365616 (99.1%)	CLCuKoV-Bur (JF502353) (99.1%)	2.998 × 10^−60^	4.847 × 10^−60^	2.748 × 10^−41^	1.113 × 10^−19^	6.866 × 10^−20^	1.271 × 10^−23^	1.067 × 10^−10^
MK357257	379	887	CLCuMuV-PK EU365616 (99%)	CLCuKoV-Bur (JF502353) (99.8%)	2.998 × 10^−60^	4.847 × 10^−60^	2.748 × 10^−41^	1.113 × 10^−19^	6.866 × 10^−20^	1.271 × 10^−23^	1.067 × 10^−10^
MK357258	376	887	CLCuMuV-PK EU365616 (98.8%)	CLCuKoV-Bur (JF502353) (99.6%)	2.998 × 10^−60^	4.847 × 10^−60^	2.748 × 10^−41^	1.113 × 10^−19^	6.866 × 10^−20^	1.271 × 10^−23^	1.067 × 10^−10^
KX656806	357	887	CLCuMuV-PK EU365616 (98.5%)	CLCuKoV-Bur (JF502353) (99.2%)	2.998 × 10^−60^	4.847 × 10^−60^	2.748 × 10^−41^	1.113 × 10^−19^	6.866 × 10^−20^	1.271 × 10^−23^	1.067 × 10^−10^
KX656809	379	880	CLCuMuV-PK EU365616 (98.3%)	CLCuKoV-Bur (JF502353) (99.8%)	2.998 × 10^−60^	4.847 × 10^−60^	2.748 × 10^−41^	1.113 × 10^−19^	6.866 × 10^−20^	1.271 × 10^−23^	1.067 × 10^−10^
KX656810	357	880	CLCuMuV-PK EU365616 (98.5%)	CLCuKoV-Bur (JF502353) (98.7%)	2.998 × 10^−60^	4.847 × 10^−60^	2.748 × 10^−41^	1.113 × 10^−19^	6.866 × 10^−20^	1.271 × 10^−23^	1.067 × 10^−10^
KY120359	328	887	CLCuMuV-PK EU365616 (97.3%)	CLCuKoV-Bur (JF502353) (99.6%)	2.998 × 10^−60^	4.847 × 10^−60^	2.748 × 10^−41^	1.113 × 10^−19^	6.866 × 10^−20^	1.271 × 10^−23^	1.067 × 10^−10^
KY120360	328	887	CLCuMuV-PK EU365616 (98.5%)	CLCuKoV-Bur (JF502353) (99.5%)	2.998 × 10^60^	4.847 × 10^−60^	2.748 × 10^−41^	1.113 × 10^−19^	6.866 × 10^−20^	1.271 × 10^−23^	1.067 × 10^−10^
KY120361	328	887	CLCuMuV-PK EU365616 (99.1%)	CLCuKoV-Bur (JF502353) (99.8%)	2.998 × 10^60^	4.847 × 10^−60^	2.748 × 10^−41^	1.113 × 10^−19^	6.866 × 10^−20^	1.271 × 10^−23^	1.067 × 10^−10^
MF184923	328	887	CLCuMuV-PK EU365616 (99.3%)	CLCuKoV-Bur (JF502353) (99.8%)	2.998 × 10^−60^	4.847 × 10^−60^	2.748 × 10^−41^	1.113 × 10^−19^	6.866 × 10^−20^	1.271 × 10^−23^	1.067 × 10^−10^
MG373551	328	887	CLCuMuV-PK EU365616 (99.4%)	CLCuKoV-Bur (JF502353) (99.6%)	2.998 × 10^−60^	4.847 × 10^−60^	2.748 × 10^−41^	1.113 × 10^−19^	6.866 × 10^−20^	1.271 × 10^−23^	1.067 × 10^−10^
MG373556	328	887	CLCuMuV-PK EU365616 (99.4%)	CLCuKoV-Bur (JF502353) (99.6%)	2.998 × 10^−60^	4.847 × 10^−60^	2.748 × 10^−41^	1.113 × 10^−19^	6.866 × 10^−20^	1.271 × 10^−23^	1.067 × 10^−10^

One common recombination event is identified in which CLCuMuV-PK (**EU365616**) and CLCuKoV-Bur (**JF502353**) were major and minor parents. Beginning and ending breakpoints of recombination breakpoints and average *P-*value of seven methods are mentioned here. Green and pink highlighted colors are for isolates of Pakistan and India, respectively.

### Determination of specific mutations in genic and intergenic regions of CLCuMuV-Raj associated with the third epidemic of CLCuD

When we compared the protein sequences of the recently identified isolates with protein sequences of previously reported CABs from this region, we found the specific mutations at specific positions in different proteins of distinct CLCuMuV strain identified in current study which were mostly substitutions with other amino acids. Moreover, in intergenic region, we also identified a deletion of 15 base-pair fragment at a specific position. Details of all the mutations identified in genes and intergenic region of CLCuMuV are given in Table 4.

**Table 4:** mutations detected in different genes/protein and intergenic region of distinct strain of CLCuMuV (associated with third epidemic of CLCuD)

Sr. No	Gene/protein	Most commonly identified mutations in distinct strain of CLCuMuV
1.	V1	G24D, T28V or T28A, S29C, Q41A, Q42K, and T45A
2.	V2	A73P, E75Q, H81R or H81C, Q82V or Q82E, K93Q or K93H, T94S or T94A, T95K, G95N, G96S, D98G, K99E, Y102H, and E105K.
3.	C1	P176L, V197E, A199V, A200S, N211H, S262V, and T263D
4.	C2	D9N, D89N, H93Y, A125P, and K132Q
5.	C3	E22A, Q76H, and H134Y
6.	C4	No specific mutations
7.	C5	Y53D, D62N, P125G, C128R, L132C, C134G, G145R or G145H, V146I, A149V, Q152E, and P154A.
8.	–	Intergenic region has deletion of 15 bp fragment, i.e. TACCATTAACACTTG

### qRT-PCR for relative levels of viruses and associated satellites

The results of qRT-PCR are shown in Fig. 3(D). Interestingly, OELCV was absent in two samples and its amplification was found in one sample with very low viral titer (amplified after 32 PCR cycles). As it was not showing significant quantity that is supporting the presence of few molecules of this species in NGS data.

### Screening of samples using CP primers of CLCuMuV-Raj strain

To confirm the widespread occurrence of CLCuMuV-Raj strain, 40 clones of the CP were sequenced. All the sequences of the CP showed the presence of this distinct strain of CLCuMuV in all symptomatic samples from different major cotton growing areas of the Punjab province of Pakistan. All isolates of CP sequences are submitted in NCBI and are available under accession numbers MT037407–MT037446.

## Discussion

Cotton, which is the most important cash crop for the Indian subcontinent, is badly affected by CLCuD for the last three decades. CLCuD has passed through two major epidemics in this region with distinct begomoviruses associated with the disease. Although at the end of 1990s this disease apparently disappeared due to development and adaptation of disease resistant cotton cultivars, but in the early 2000s, this disease reappeared, and was associated with the recombinant strain CLCuKoV-Bur. It was also associated with a recombinant CLCuMuB which is currently known as CLCuMuB^Bur^. This strain remained dominant till 2014 but in the year 2015, multiple begomoviruses which were associated with the first epidemic were found in cultivated cotton in Pakistan and CLCuMuV was rebound to CLCuD and exclusively detected from India in the same year [21, 23]. In both studies, CLCuKoV-Bur was not identified in cultivated cotton. Sattar *et al.* [24] predicted the third epidemic of CLCuD in this region in 2017–18 on the basis of the statistical data of environmental changes and the diversity of the begomoviruses associated with this disease. They predicted the years from 2014 to 2016, similar to the few years of second pre-epidemic time period when disease complex was evolving and apparently cotton production was recovered to the first pre-epidemic level. Interestingly, our field data of virus hotspots support their prediction. We found fewer hotspots of CLCuD from 2014 to 2016 in Punjab, Pakistan. In the years 2017 and 2018, major cotton growing areas of Punjab, Pakistan were badly affected by CLCuD. The overall disease incidence of 24% and 29% was recorded in 2017 and 2018, respectively, from Punjab, Pakistan. Virus hotspots spread all over the major cotton growing areas of Punjab (Fig. 1). So, it was important to precisely determine genetic diversity associated with this new outbreak in the region. Previously two molecular techniques were adopted to clone the begomoviruses and associated satellites; 1) PCR amplification using universal primers, 2) Restriction cloning (by digesting the enriched viral genomes using different restriction enzymes and their cloning), and finally their Sanger sequencing. Recently, NGS, using the Ilumina platform, of restricted RCA product then reference based or novel assembly of the sequenced reads was carried out. All the drawbacks of these previously adopted techniques are discussed in Mehta *et al.* [25] where they introduced the new method for sequencing such virus molecules. We adopted this new method with some modifications and provided solutions of some technical issues in analyzing the complex genetic diversity. We used this method for the first time to explore the genetic diversity associated with CLCuD, in future this methodology can be adopted to explore the genetic diversity associated with other such complex diseases. Findings of the current study are very interesting because the same strain of CLCuMuV was reported from different locations (Bernala, Punjab, and New Delhi) in India since the 2015. As in the same year, same strain of CLCuMuV was identified from Vehari, Pakistan with multiple begomoviruses. Thus, it is difficult to exactly predict the origin of this distinct strain of CLCuMuV. One of the most important indications for its origin from India is that its most similar strain, i.e. CLCuMuV-Raj originated from India and only its single isolate was found from Pakistan in 2009 in cotton wild species. But at the same time a strong recombination event identified in all isolates of this distinct clade showed that it originated from recombination between CLCuMuV-PK strain (EU365616) identified from Pakistan [50] and CLCuKoV-Bur (JF502353) identified from India [51]. Whatever is the origin of this distinct strain it is now widespread in both countries. Its recent identification from a hollyhock plant in New Delhi, India shows that it is widespread in other hosts as well. In Punjab, Pakistan, the Asia II-1 species of whitefly is dominant and recently it has been shown that Asia II-I very efficiently transmits CLCuMuV whereas Middle-east Asia Minor-1 (MEAM1) efficiently transmits Tomato yellow leaf curl virus (TYLCV) [52]. Thus, the widespread of CLCuMuV with Asia II-I in Punjab is not surprising and in a recent survey of whitefly from Pakistan has also shown the dominance of CLCuMuV-Raj strain in whitefly collected from cotton in major cotton growing areas of Punjab [53].

We determined the specific mutations in genes and intergenic region of CLCuMuV associated with the third epidemic of CLCuD. The finding of these mutations will be helpful in understanding the molecular interaction of virus with the host and vector. To the best of our knowledge, this is the first exclusive study which specifically determined the disease complex associated with its third epidemic in this region. The findings of this study will be helpful in understanding the molecular mechanism of CLCuD as well as timely development of the durable and broad-spectrum resistant cultivars of cotton. Finally, the NGS sequencing method used in current and its downstream analysis for precise identification of genetic diversity may be helpful for the exploration of other such complex diseases.

### Conclusion and prospects

The third epidemic of CLCuD is started not only in Punjab, Pakistan but similar is expected from India because situation is similar since 2015 in both countries. Interestingly, same disease complex is widespread in both countries. It is an alarming situation for the cotton production in both countries. Hopefully, this report will be helpful for the scientists of both countries to timely think about the next severe disaster which may be accompanied with this epidemic of CLCuD in the Indian sub-continent. Therefore, it is important to timely develop the virus resistant cultivars of cotton whether using breeding technologies or the latest genetic engineering techniques. Moreover, it is also most important to deeply understand the molecular mechanism of CLCuD so that it could be possible to break the virus–host–vector compatibility and get the durable resistance against this disease.

## Ethical approval

This research work did not involve any human or animal concern which requires ethical approval.

## Consent for publication

Data of CLCuD incidence was taken from department of extension, Govt. of Punjab, Pakistan and its consent for publication was given by its Director General (DG).

## Data availability

Sequences of all the isolates of begomoviruses and satellites identified in current study were deposited in NCBI’s Genbank and are available under the accession numbers mentioned here. Begomoviruses: MK357244-MK357258, MT037028-MT037033, and MT037052-MT037060. Betasatellites: MK357271-MK357285, MT037034-MT037042, and MT037061-MT037063. Alphasatellites: MK357286-MK357327 and MT037043- MT037051. Viral CPs: MT037407-MT037446.
